# Entry Pathways of Herpes Simplex Virus Type 1 into Human Keratinocytes Are Dynamin- and Cholesterol-Dependent

**DOI:** 10.1371/journal.pone.0025464

**Published:** 2011-10-12

**Authors:** Elena Rahn, Philipp Petermann, Mei-Ju Hsu, Frazer J. Rixon, Dagmar Knebel-Mörsdorf

**Affiliations:** 1 Center for Biochemistry, University of Cologne, Cologne, Germany; 2 Department of Dermatology, University of Cologne, Cologne, Germany; 3 MRC-University of Glasgow Center for Virus Research, Glasgow, United Kingdom; 4 Institute of Oral Biology, National Yang-Ming University, Taipei, Taiwan; University of Minnesota, United States of America

## Abstract

Herpes simplex virus type 1 (HSV-1) can enter cells via endocytic pathways or direct fusion at the plasma membrane depending on the cell line and receptor(s). Most studies into virus entry have used cultured fibroblasts but since keratinocytes represent the primary entry site for HSV-1 infection in its human host, we initiated studies to characterize the entry pathway of HSV-1 into human keratinocytes. Electron microscopy studies visualized free capsids in the cytoplasm and enveloped virus particles in vesicles suggesting viral uptake both by direct fusion at the plasma membrane and by endocytic vesicles. The ratio of the two entry modes differed in primary human keratinocytes and in the keratinocyte cell line HaCaT. Inhibitor studies further support a role for endocytosis during HSV-1 entry. Infection was inhibited by the cholesterol-sequestering drug methyl-β-cyclodextrin, which demonstrates the requirement for host cholesterol during virus entry. Since the dynamin-specific inhibitor dynasore and overexpression of a dominant-negative dynamin mutant blocked infection, we conclude that the entry pathways into keratinocytes are dynamin-mediated. Electron microscopy studies confirmed that virus uptake is completely blocked when the GTPase activity of dynamin is inhibited. *Ex vivo* infection of murine epidermis that was treated with dynasore further supports the essential role of dynamin during entry into the epithelium. Thus, we conclude that HSV-1 can enter human keratinocytes by alternative entry pathways that require dynamin and host cholesterol.

## Introduction

Herpes simplex virus type 1 (HSV-1) enters its human host via epithelia of mucosa, skin or cornea where keratinocytes represent the primary entry site. Cellular entry of HSV-1 involves multiple steps. Initial virus-cell contact is mediated by HSV-1 envelope glycoproteins gC and/or gB with cell surface heparan sulfate proteoglycans which facilitate subsequent binding to coreceptors. The viral envelope glycoprotein gD serves as the major virus ligand for all known HSV coreceptors and the best studied gD coreceptor is the immunoglobulin-like cell-cell adhesion molecule nectin-1 (named HveC) [1]. Depending on the cell line HSV-1 can enter cells either by direct fusion of the viral envelope with the plasma membrane or by endocytic pathways [2], [3], [4], [5] which can be both pH-dependent and pH-independent [6]. Entry into neurons and Vero cells can occur via fusion at the plasma membrane at neutral pH while fusion with HeLa and CHO cells involves pH-dependent endocytosis, and fusion with C10 (B78-H1 mouse melanoma expressing nectin-1) cells involves pH-independent endocytosis. Interestingly, expression of nectin-1 in CHO cells correlates with endocytic uptake while expression of PILRα (paired immunoglobulin-like type 2 receptor α) in CHO cells points to HSV-1 uptake via fusion suggesting that the entry pathway into the same cell line depends on the cellular entry coreceptor used [7]. Furthermore, the same receptor may initiate different entry pathways, depending on the cell in which it is expressed. When expressed in the J1.1-2 cell line, nectin-1 mediates entry that is not blocked by endosome acidification inhibitors, however, nectin-1 mediated entry into CHO cells is dependent on endosome acidification [2]. After additional overexpression of αvβ3-integrin, HSV-1 entry in J1.1-2 nectin-1 cells is cholesterol- and dynamin-independent whereas cholesterol and dynamin play a role in CHO-nectin-1 expressing cells [8]. A phagocytosis-like uptake in which dynamin-mediated processes have been implicated, has been also suggested for CHO-nectin-1 expressing cells [9]. Dynamin is a multidomain GTPase that controls several distinct endocytic pathways, with the clathrin-mediated endocytosis being the best studied [10]. Dynamin plays a direct role in catalyzing membrane fission. During clathrin-mediated endocytosis dynamin forms a helical polymer around the vesicle neck and, upon GTP hydrolysis, mediates the fission of the vesicle from the plasma membrane [11]. Recent studies have also implicated dynamin in further cellular processes such as regulation of actin assembly and reorganization via its interactions with many actin-binding proteins [12], [13]. Furthermore, dynamin can function in the process of fusion pore expansion and postfusion events in exocytosis [14], [15].

HSV-1 seems to be capable of using a variety of entry mechanisms that may reflect an adaptation to differences in its target cells. The goal of this study was to characterize the HSV-1 entry mechanisms into human keratinocytes since little is known about this entry portal in the human host. There has been one report that HSV-1 may enter keratinocytes via a pH-dependent endocytic pathway [4]. The authors showed that treatment with agents that elevate endosomal pH inhibits entry, and cellular tyrosine kinase activity is selectively required for efficient entry by the low-pH, endocytic pathway [4].

Our results suggest that HSV-1 enters human keratinocytes both by direct fusion of virions at the cell surface and by an endocytic pathway. As dynamin is an important player during endocytic uptake we addressed its impact during entry into keratinocytes. Interestingly, dynamin inhibitors blocked infection by interfering with penetration of the virions at the plasma membrane which in turn inhibited both fusion at the plasma membrane and vesicle formation. Furthermore, we provide the first evidence that host cholesterol plays an important role during entry into keratinocytes.

## Results

### Uptake of HSV-1 into human keratinocytes

We infected HaCaT cells representing undifferentiated human keratinocytes, and primary human epidermal keratinocytes to analyze the mode of virus uptake using electron microscopy. All studies were performed with high MOI (200 or 1500 PFU/cell) to achieve infection of all cells at rather high cell density. Primary keratinocytes were cultured in calcium-reduced medium to minimize cell-cell contacts and thereby enhance infectivity.

**Figure 1:**
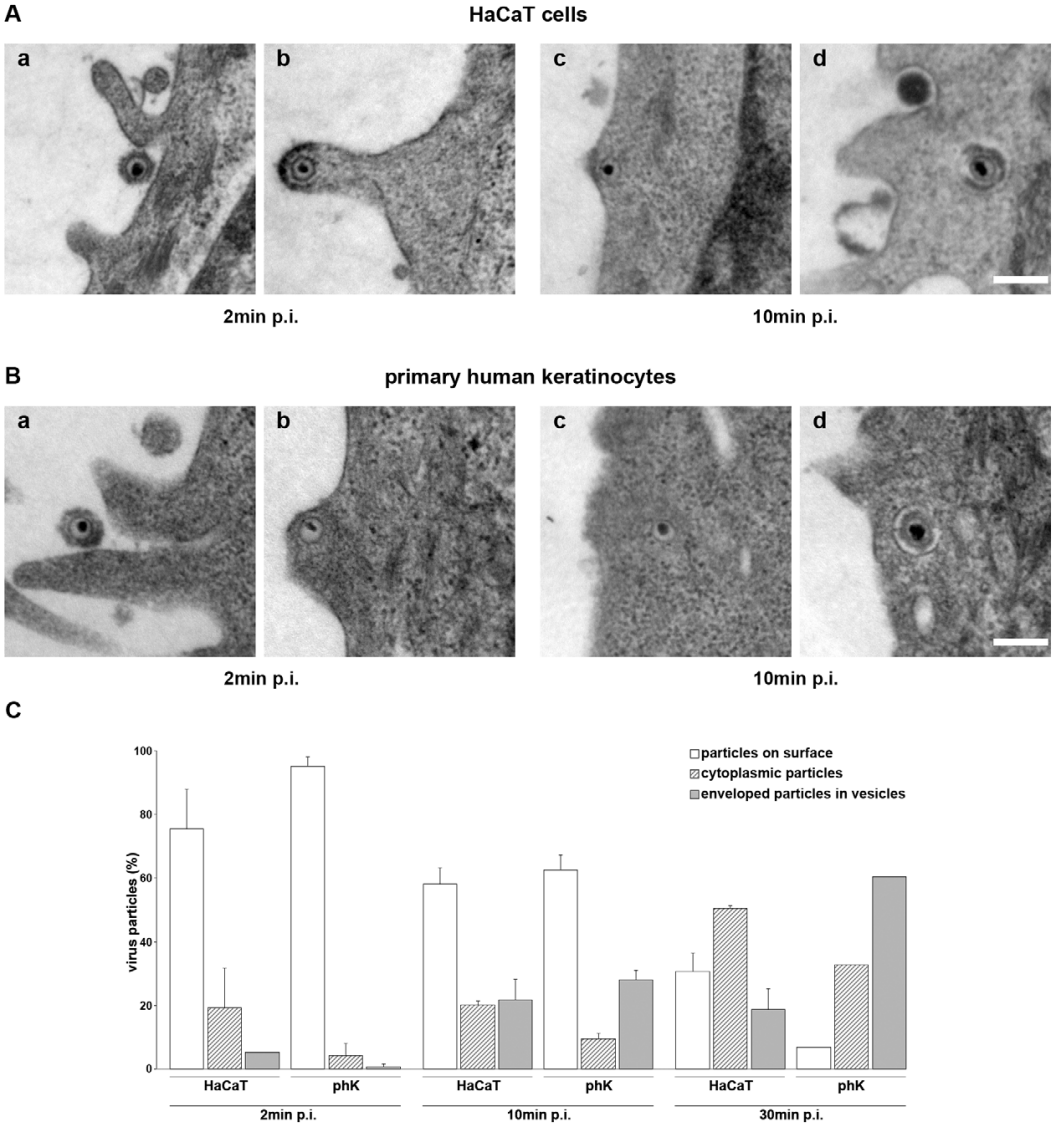
Uptake of HSV-1 into human keratinocytes. (A) HaCaT cells or (B) primary human keratinocytes (phK) were incubated with HSV-1 at 200 PFU/cell (A, a, b), or 1500 PFU/cell (A, c, d; B, a–d) for 1 h at 4°C to allow attachment followed by incubation at 37°C to allow uptake. Cells were fixed and prepared for electron microscopy at 2, 10, or 30 min p.i. (A, B) Particles on the cell surface (a), free cytoplasmic capsids (b, c) and enveloped particles in vesicles (d) are shown at 2 and 10 min p.i. Bar, 0.2 µm. (C) In two independent experiments 133, 138, and 109 particles in total were evaluated at 2, 10, and 30 min, respectively, in HaCaT cells, and 159 (at 2 min) and 254 (at 10 min) were analyzed in primary human keratinocytes. The percentages of particles on the surface, cytoplasmic capsids and enveloped particles in vesicles are given at 2, 10, and 30 min p.i. Results are mean ± standard deviation values. In primary keratinocytes 58 particles in total were analyzed in one experiment at 30 min p.i.

At 2 min p.i. most virions were observed at the cell surface of HaCaT cells while 24% of the virus particles were internalized. Interestingly, 5% were found in vesicles and 19% were detectable as free capsids underneath the plasma membrane (Fig. 1A, C). The same ratio of free capsids and enveloped particles in vesicles was present in primary keratinocytes although at much lower percentages suggesting that virus uptake was initially delayed as compared to HaCaT cells (Fig. 1B, C). At 10 min p.i. the same quantity of either free capsids or of particles in vesicles were observed in HaCaT cells while by 30 min p.i. free capsids were more abundant than particles in vesicles (Fig. 1C). In contrast, the percentage of free capsids in primary keratinocytes was significantly lower than the percentages of particles in vesicles at 10 and 30 min p.i. (Fig. 1C). The results suggest that uptake of HSV-1 into keratinocytes can occur via both direct fusion of the viral envelope with the plasma membrane and via an endocytic pathway. Interestingly, the ratio between the two uptake modes differed in primary cells compared with HaCaT cells suggesting that endocytic uptake is more pronounced in primary keratinocytes.

We assume that the free capsids observed in the cytoplasm at 2 min p.i. were released following very rapid fusion at the plasma membrane. Many of the capsids observed at 2 and 10 min p.i. were located just underneath a region of the plasma membrane with a distinctive staining pattern resembling that of the viral envelope (Fig. 1 A, b, c; B, b). This is highly suggestive of a direct fusion process. However, some of the free capsids observed at 10 and 30 min p.i. might be released from endosomes.

### Role of endocytic pathways

The potential role of endosomal HSV-1 uptake into keratinocytes was analyzed by inhibitor studies. Successful infection in individual cells was visualized by staining with an antibody directed against the viral immediate-early protein ICP0. The cellular localization of ICP0 passes through distinct phases during early infection, in which ICP0 in nuclear foci indicates an early stage of viral gene expression followed by a cytoplasmic ICP0 relocalization [16]. Therefore, a reduction in the amount of cytoplasmic ICP0 following drug treatment suggests delayed infection, although we cannot exclude the possibility that any one drug could have an effect on export of ICP0 from the nucleus. Subconfluent cells were infected with 20 PFU/cell which led to ∼80–90% of HaCaT cells and ∼40–60% of primary keratinocytes becoming infected based on ICP0 expression visualized at 2 h p.i.

**Figure 2:**
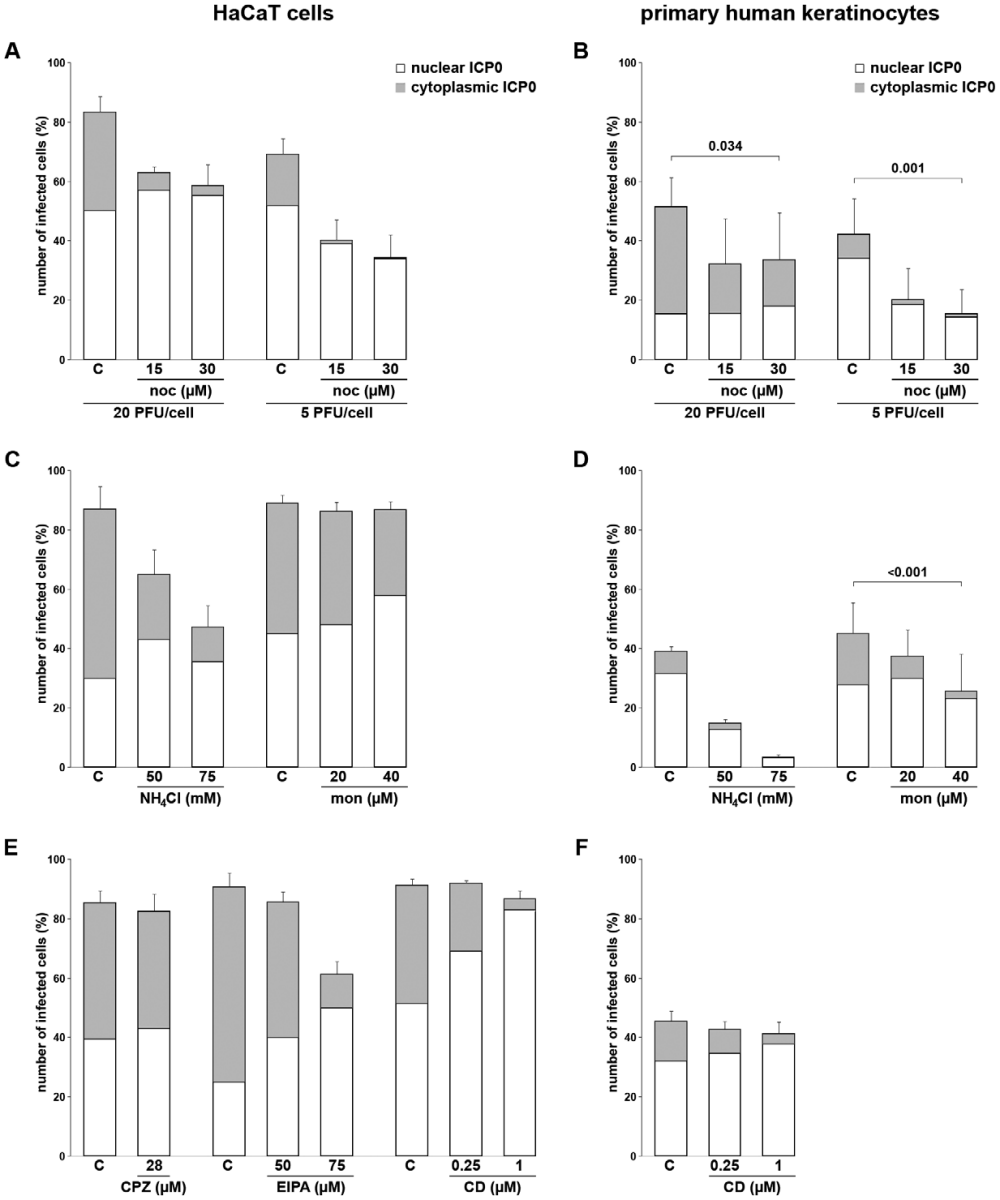
HSV-1 infection of keratinocytes pretreated with inhibitors against microtubules, actin, clathrin, sodium/proton exchanger or with lysosomotropic agents. HaCaT cells or primary human keratinocytes were pretreated with the indicated concentrations of drugs or corresponding solvent controls (C) for 30 min followed by infection with HSV-1 in the presence of the drugs. All infections were at 20 PFU/cell apart from the nocodazole (noc)-treated cells which were infected both at 20 and 5 PFU/cell. All cells were fixed at 2 h p.i. and stained with mouse anti-ICP0. The number of ICP0-expressing cells was determined in at least three independent experiments. The location of ICP0 expression was recorded as only in the nucleus (nuclear ICP0) or in both nucleus and cytoplasm (cytoplasmic ICP0) representing early and early-late phases during infection, respectively. The percentages of infected cells are shown after treatment with nocodazole (noc) in panels A and B, with NH_4_Cl and monensin (mon) in C and D, and with chlorpromazine (CPZ), EIPA and cytochalasin D (CD) in E and F. Results are mean ± standard deviation values. P-values (pair-t-test) are given above the diagram (B, D).

When the microtubule-depolymerizing drug nocodazole which inhibits trafficking from early to late endosomes [17], was added prior to infection, the number of infected HaCaT cells was reduced in a concentration-dependent manner from 83% to 58%. The reduction was more marked when the MOI was lowered from 20 to 5 PFU/cell. In addition, the proportion of infected cells with cytoplasmic ICP0 decreased from 40% to 6% (20 PFU/cell) in drug-treated cells suggesting a delay of infection during the early phase (Fig. 2A). When primary human keratinocytes were treated with the same amounts of nocodazole, infection was reduced from 51% to 33% at 20 PFU/cell and from 42% to 15% at 5 PFU/cell (Fig. 2B). Thus, our results support a role for trafficking via the microtubule network in the entry pathway in keratinocytes. However, this experiment does not distinguish between trafficking of free capsids or trafficking of vesicles containing enveloped particles from early to late endosomes.

Cells were treated with lysosomotropic agents to address the impact of endosomal acidification during HSV-1 entry into keratinocytes. While treatment of HaCaT cells with the carboxylic ionophore monensin (40 µM) did not interfere with infectivity, addition of the weak base ammonium chloride (NH_4_Cl) reduced the level of infection from 87% to 47% of cells in a concentration-dependent manner (Fig. 2C). In addition, the decrease in cytoplasmic ICP0 localization in NH_4_Cl-treated HaCaT cells suggested delayed early infection (Fig. 2C). In primary keratinocytes the concentration-dependent effect of NH_4_Cl was much stronger. We observed a reduction in the number of infected cells from 39% to 3% in the presence of 75 mM NH_4_Cl (Fig. 2D). In contrast to HaCaT cells monensin-treatment of primary keratinocytes also decreased the number of infected cells from 45% to 26% (Fig. 2D). There was a concomitant reduction in the number of infected cells with cytoplasmic ICP0 in all drug-treated primary cells. Taken together, the results demonstrate that NH_4_Cl produced a limited and monensin no reduction in virus infectivity in HaCaT cells, while both agents had much greater effects in primary keratinocytes.

**Figure 3:**
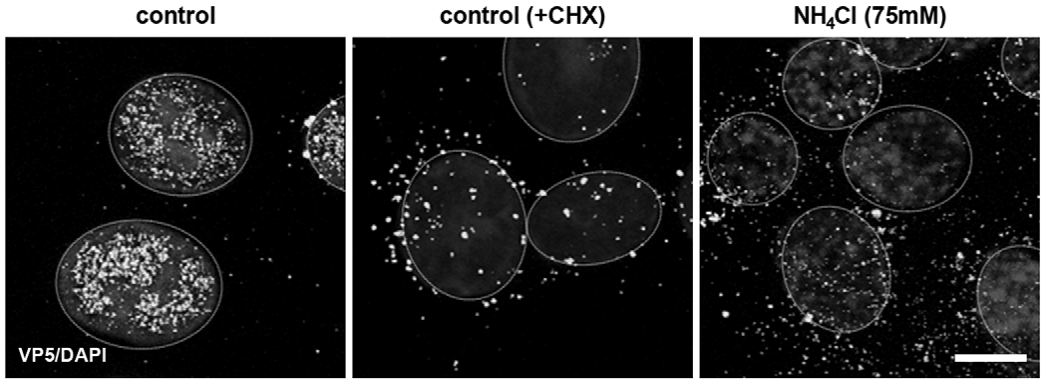
Localization of HSV-1 particles in NH_4_Cl-treated primary human keratinocytes cells. Untreated cells (control) or cells pretreated with 75 mM NH_4_Cl were incubated with 100 PFU/cell of HSV-1 for 2 h at 4°C followed by incubation at 37°C. After 2 h at 37°C cells were either treated with 0.1 mM cycloheximide or medium was replaced. After 5 h at 37°C cells were fixed and stained with mouse anti-VP5, visualized with AF488-conjugated antimouse (Molecular Probes). Confocal projections are shown and nuclei stained with 4′, 6′-diamidino-2-phenylindole (DAPI) are circled. Bar, 8 µm.

Control experiments indicated that the reduction in infectivity by NH_4_Cl was reversible when the weak base was removed just prior to infection (data not shown). Based on DAPI staining of the cell nucleus, the chromatin seemed to be changed in the presence of NH_4_Cl. To exclude the possibility that the observed effects were due to a transcriptional block, GFP-expressing plasmids were transfected which demonstrated unchanged GFP expression in NH_4_Cl-treated HaCaT cells (data not shown). Furthermore, we analyzed the localization of HSV-1 in NH_4_Cl-treated primary keratinocytes to confirm that the elevated pH in intracellular compartments interferes with the delivery of capsids to the nuclear periphery as recently described [4]. Since in untreated control cells nuclear accumulation of newly synthesized capsid protein VP5 was observed already early during infection, we inhibited protein expression by cycloheximide to visualize input capsids in close proximity to the nucleus (Fig. 3). In contrast, input capsids were widely dispersed in NH_4_Cl-treated cells and localized only rarely to the nuclear periphery (Fig. 3). In summary, we conclude that endosomal acidification contributes to HSV-1 infection of HaCaT cells and may play a more prominent role in primary keratinocytes.

To gain further insights into potential endocytic pathways, keratinocytes were treated with chlorpromazine which leads to misassembly of clathrin-coated pits by inhibiting the assembly of the clathrin adaptor protein AP2 [18]. Since treatment of HaCaT cells with 28 µM chlorpromazine had no influence on infectivity (Fig. 2E), we infer that clathrin-mediated endocytosis does not contribute to HSV-1 entry. We then treated HaCaT cells with the sodium-proton exchange inhibitor 5-(N-Ethyl-N-isopropyl)amiloride (EIPA) which is used as specific inhibitor of macropinocytosis [19]. The number of infected cells dropped from 90% to 61% when 75 µM EIPA was added prior to infection (Fig. 2E). Recent studies indicate that EIPA has significant effects on various endocytic processes such as relocalization of early and late endosomes [20]. Thus, the reduction in infectivity caused by EIPA may simply reflect the interference with an endocytic pathway. Since macropinosome formation involves filamentous actin (F-actin), keratinoyctes were treated with cytochalasin D (CD) which blocks actin polymerization at the barbed ends of F-actin. In both HaCaT cells and primary keratinocytes CD treatment did not reduce the number of infected cells, although, early infection seemed to be delayed in a concentration-dependent manner (Fig. 2E, F). These results suggest that macropinocytosis is not favored during HSV-1 entry into either HaCaT cells or primary keratinocytes.

In summary, the inhibitor studies suggest that the microtubule network and endosomal acidification contribute to the HSV-1 entry pathway in keratinocytes. The ion-transport inhibitor EIPA had some inhibiting effect on infectivity whereas disassembly of actin filaments correlated with only a minor effect on infection which does not support a major role for the macropinocytic pathway but indicates involvement of other endocytic processes. Taking these results together we conclude that endocytic pathways play a role during HSV-1 entry into keratinocytes.

### Impact of cholesterol

**Figure 4:**
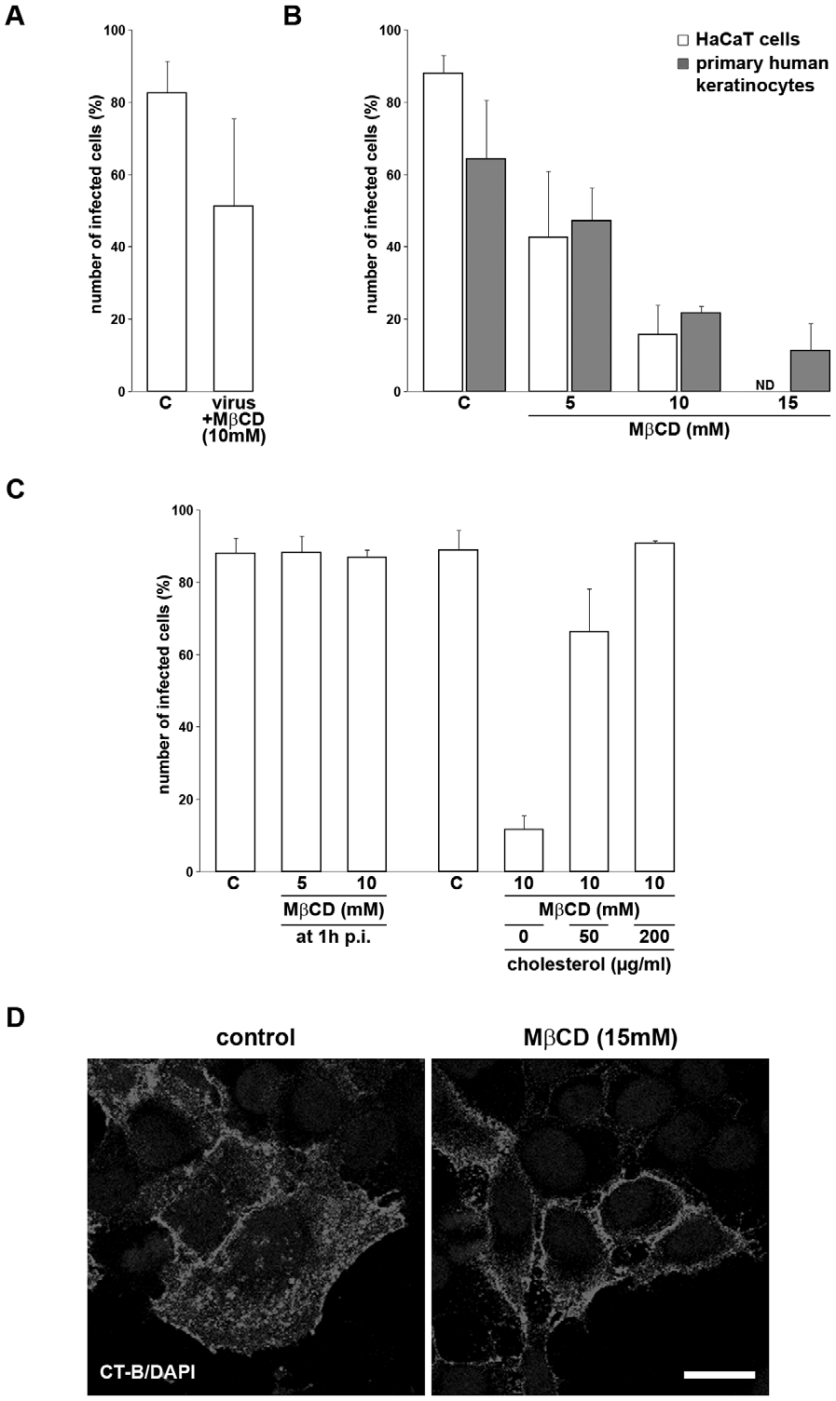
HSV-1 infection of keratinocytes upon depletion of cholesterol. (A) HaCaT cells were infected with HSV-1 pretreated with MβCD (10 mM) for 30 min at room temperature or with untreated HSV-1 at 20 PFU/cell. (B) HaCaT cells or primary human keratinocytes were pretreated with the indicated concentrations of MβCD for 30 min at 37°C. After washing with medium cells were infected with HSV-1 at 20 PFU/cell. As a control (C), cells were infected in the absence of the drug. (C) HaCaT cells were infected with HSV-1 at 20 PFU/cell. At 1 h p.i. 5 or 10 mM MβCD was added to the cells. In addition, HaCaT cells were pretreated with 10 mM MβCD for 30 min, and 0, 50 or 200 µg/ml cholesterol was added prior to infection. (A, B, C) At 2 h p.i. cells were fixed, stained with mouse anti-ICP0 and the number of ICP0-expressing cells was determined in at least three independent experiments. Results are mean ± standard deviation values. (D) HaCaT cells were untreated or pretreated with 15 mM MβCD for 30 min and AF594-conjugated cholera toxin B (CT-B) (5 µg/ml) was added. Cells were fixed and stained with DAPI. Bar, 20 µm.

Many endocytic pathways require cholesterol-rich lipid rafts for their function. The essential role of cholesterol for HSV-1 entry into Vero cells and mouse melanoma cells expressing either nectin-1 or HVEM has been shown [21]. We treated keratinocytes with methyl-β-cyclodextrin (MβCD) which depletes cholesterol from the plasma membrane to address the requirement for cholesterol for HSV-1 entry in keratinocytes [22], [23], [24]. The functionality of MβCD in keratinocytes was initially confirmed by visualizing the uptake of cholera toxin B, a glycosphingolipid-binding ligand that is known to be internalized by caveolae-mediated and lipid-raft-dependent endocytosis [25], [26]. When HaCaT cells were pretreated with 15 mM MβCD for 30 min cholera toxin B uptake was efficiently blocked as visualized by the loss of cytoplasmic cholera toxin B (Fig. 4D). Upon HSV-1 infection a concentration-dependent inhibition of infectivity was visible in both MβCD-treated HaCaT cells and primary keratinocytes. Upon pre-treatment with 10 mM MβCD the number of infected cells was reduced from 88% to 16% in HaCaT cells and from 64% to 22% in primary keratinocytes (Fig. 4B). Prior to infection MβCD was removed to avoid any depleting effect on cholesterol in the viral envelope. Control experiments indicate that preincubation of HSV-1 particles with 10 mM MβCD reduced infectivity (Fig. 4A). This observation is in agreement with previous results demonstrating a reduced infection rate of pseudorabies virus (PrV) when viral cholesterol was depleted with MβCD [27]. When 10 mM MβCD was added to the cells at 1 h p.i., we observed no effect on the number of infected cells (Fig. 4C) suggesting that depletion of cholesterol in the plasma membrane interferes directly with HSV-1 uptake and that MβCD does not disturb subsequent steps during early infection. In addition, we addressed whether cholesterol depletion was reversible by giving 50 or 200 µg/ml cholesterol to MβCD-treated cells. Following infection we observed a concentration-dependent increase in infectivity (Fig. 4C) demonstrating that replenishment of cholesterol restored infectivity.

When filipin which binds cholesterol was used, we observed a reduction in the number of infected primary keratinocytes, however, no effect was visible in HaCaT cells (data not shown). Since filipin also failed to inhibit cholera toxin B uptake into HaCaT cells we conclude that filipin insufficiently sequestered cholesterol in these cells.

Taken together, these results indicate that cholesterol in the plasma membrane is required for HSV-1 uptake into keratinocytes.

### Impact of dynamin

**Figure 5:**
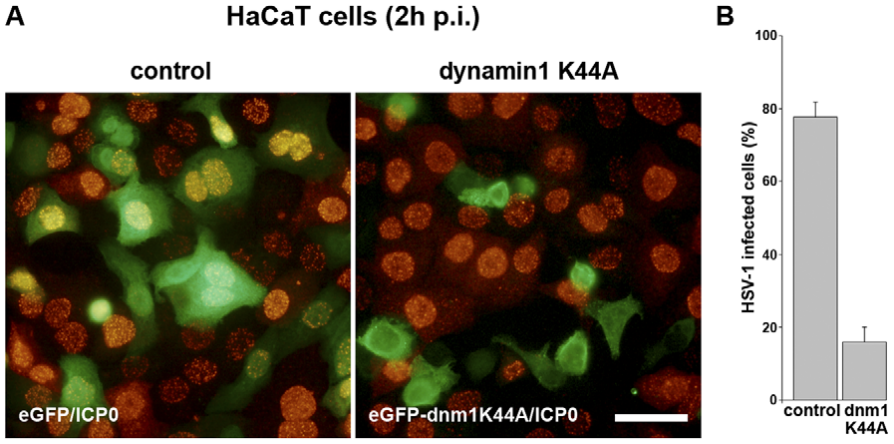
HSV-1 infection of HaCaT cells transfected with the dynamin1 mutant K44A. (A) Cells were transfected with plasmids either expressing the green-fluorescent protein (control) or the green-fluorescent protein fused to the dominant-negative dynamin1 mutant (K44A). At 22 h posttransfection the cells were infected with HSV-1 (50 PFU/cell). At 2 h p.i. cells were fixed and stained with mouse anti-ICP0 (red) visualized with AF555-conjugated anti-mouse (Molecular Probes). Bar, 40 µm. (B) The number of ICP0-expressing cells per K44A-expressing cell was determined in three independent experiments. As a control, the number of infected cells that were expressing eGFP is shown. The percentages of infected cells that were expressing the indicated plasmids are given. Results are mean ± standard deviation values.

The GTPase dynamin controls several distinct endocytic pathways [11]. To determine the role of dynamin during HSV-1 uptake, we performed overexpression, RNA interference and inhibitor studies. To overexpress the dominant-negative dynamin mutant K44A [28], HaCaT cells were transfected with plasmids expressing either the GFP-tagged dynamin mutant or GFP alone followed by infection. At 2 h p.i. we observed a reduced number of infected cells in the presence of the overexpressed dynamin mutant. Whereas 78% of the GFP-expressing cells became infected, the presence of the overexpressed dominant-negative dynamin mutant reduced the number of infected cells to 16% (Fig. 5A, B). In contrast, overexpression of wt dynamin had no inhibitory effect (data not shown).

In keratinocytes the ubiquitously expressed dynamin 2 is present, while dynamin 1 expression is restricted to neurons and dynamin 3 is expressed in lung, heart, brain and testis. When we reduced dynamin 2 expression in HaCaT cells, almost no effect on infection was observed (data not shown). Since dynamin 2 expression was only reduced by ∼80% after silencing, we conclude that the residual amount of dynamin 2 was still sufficient to allow viral entry.

**Figure 6:**
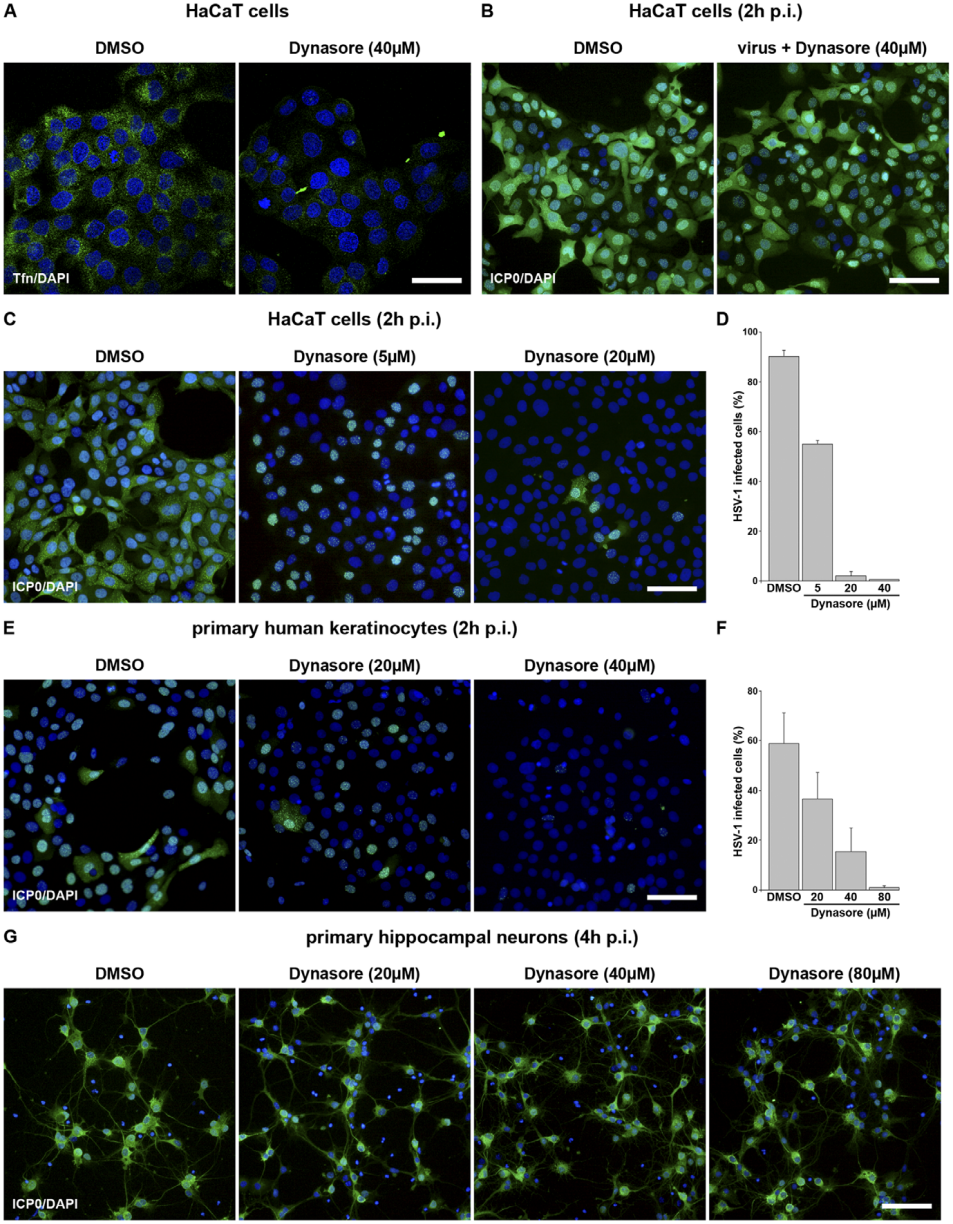
HSV-1 infection of dynasore-treated cells. All pretreatments with DMSO or dynasore were carried out for 30 min at 37°C. (A) HaCaT cells were pretreated with 0.4% DMSO or 40 µM dynasore and 50 µg/ml AF488-conjugated transferrin (Tfn) (green) was added for 15 min. Cells were fixed and stained with DAPI. Confocal projections are shown. Bar, 40 µm. (B) HSV-1 pretreated with DMSO (0.4%) or dynasore (40 µM) was used to infect HaCaT cells at 20 PFU/cell. (C, D) HaCaT cells, (E, F) primary human keratinocytes, or (G) primary hippocampal neurons were pretreated with DMSO or the indicated concentrations of dynasore followed by infection with HSV-1 at 20 PFU/cell. (B–G) At 2 h p.i. cells were fixed and costained with DAPI and mouse anti-ICP0 (green). Overlays of immunofluorescence analyses are shown. Bar, 80 µm. The number of ICP0-expressing cells was determined in (D) HaCaT cells, and (F) primary human keratinocytes in at least three independent experiments. Results are mean ± standard deviation values.

To further analyze the impact of dynamin we pretreated keratinocytes with dynasore, a small molecule inhibitor of the dynamin GTPase activity [29]. As a control we confirmed that dynamin-dependent transferrin uptake was blocked in HaCaT cells pretreated with 40 µM dynasore (Fig. 6A) [30], [31]. When dynasore was added either to HaCaT cells or primary keratinocytes a concentration-dependent inhibition of HSV-1 infection was observed. However, while 20 µM dynasore almost completely blocked infection of HaCaT cells (Fig. 6C, D), 80 µM dynasore was needed to produce the same level inhibition in primary keratinocytes (Fig. 6E, F). When dynasore (80 µM) was washed out prior to infection, no inhibitory effect on the number of infected cells was observed (data not shown). In contrast to keratinocytes, no inhibitory effect was detectable when murine hippocampal primary neurons were pretreated with up to 80 µM dynasore (Fig. 6G). These results suggest that dynamin is essential for HSV-1 entry into keratinocytes but does not play a role during entry into neurons.

To exclude any direct effects of dynasore on virus particles, we analyzed the infectivity of dynasore pretreated virions. No difference in the number of infected cells was visible when HaCaT cells were infected with untreated or dynasore-pretreated particles (Fig. 6B) confirming that dynasore interfered only with cellular functions. As a further control to rule out possible adverse actions of dynasore itself, we analyzed the effect of MiTMAB, a surface-active inhibitor that blocks dynamin's interactions with phospholipids [32]. Upon pre-treatment of HaCaT cells with MiTMAB (1–20 µM) a concentration-dependent inhibition of infection was detectable which was comparable to the effects observed in dynasore-treated cells (data not shown).

**Figure 7:**
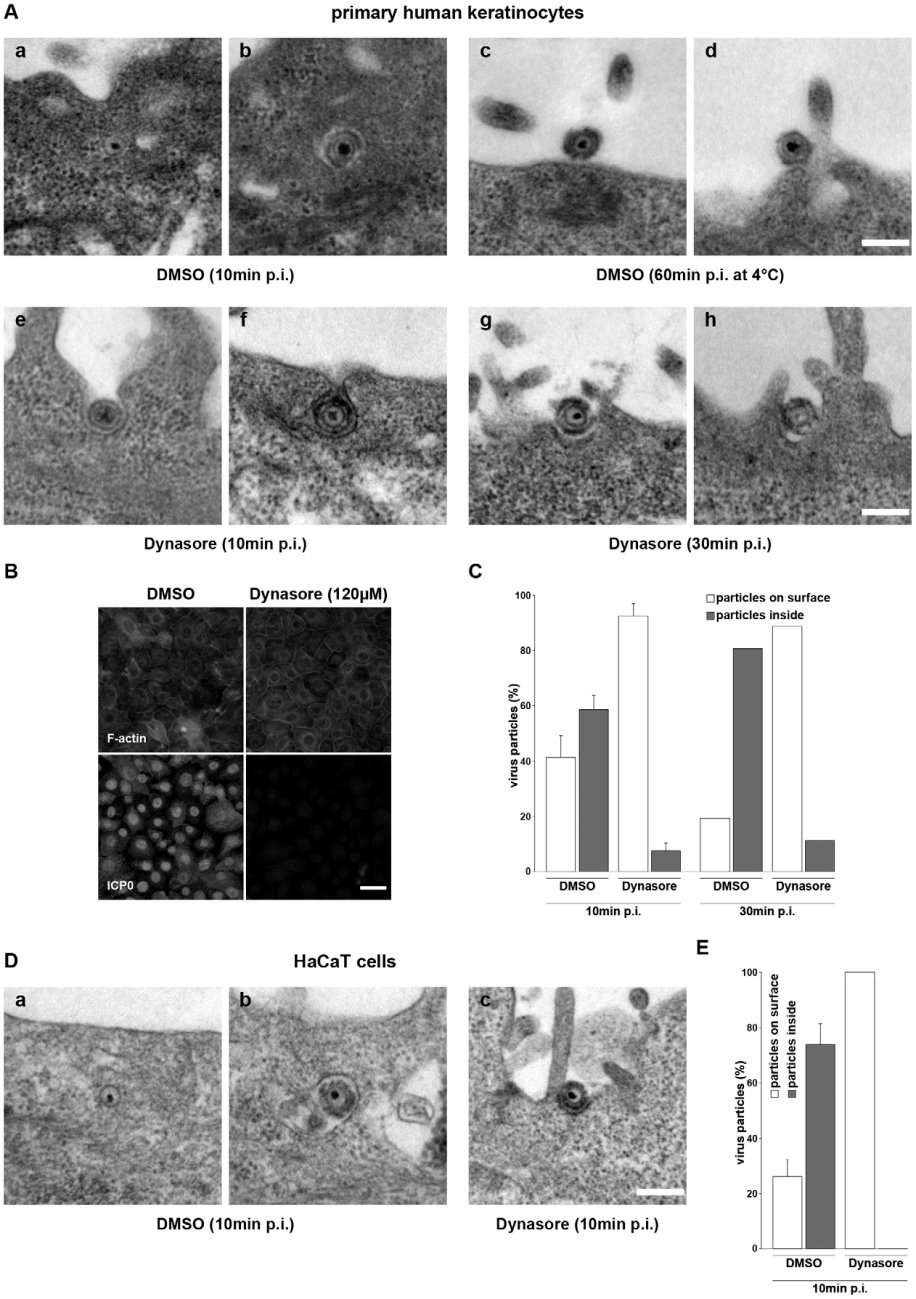
Uptake of HSV-1 into dynasore-treated keratinocytes. Primary human keratinocytes and HaCaT cells were pretreated with 120 or 80 µM dynasore, and correspondingly with 1.2% or 0.8% DMSO for 30 min at 37°C followed by 15 min at 4°C to precool the cells. Cells were incubated with HSV-1 (1500 PFU/cell) for 1 h at 4°C followed by incubation at 37°C. (A) Infected primary human keratinocytes were fixed and prepared for electron microscopy at 10 min (a, b, e, f), or 30 min at 37°C (g, h). As control, DMSO-pretreatd cells were incubated with HSV-1 (1500 PFU/cell) for 60 min at 4°C (c, d). Bar, 0.2 µm. (B) At 2 h at 37°C infected primary human keratinocytes were fixed and costained with TRITC-phalloidin (red) to visualize F-actin and mouse anti-ICP0. Single immunofluorescence analyses are shown. Bar, 40 µm. (C) Percentages of particles on surface, and particles inside including free cytoplasmic capsids and enveloped particles in vesicles are shown in DMSO- or dynasore-treated primary keratinocytes at 10 and 30 min at 37°C. In two independent experiments 108 (DMSO) and 122 (Dynasore) particles in total were evaluated for the 10 min time point and in one experiment 52 (DMSO) and 62 (Dynasore) particles were analyzed for the 30 min time point. (D) Infected HaCaT cells pretreated with DMSO or dynasore were fixed and prepared for electron microscopy at 10 min at 37°C (a–c). Bar, 0.2 µm. (E) Percentages of particles on surface, and particles inside are shown in DMSO- or dynasore-treated HaCaT cells at 10 min at 37°C. In two independent experiments 78 (DMSO) and 76 (Dynasore) particles in total were evaluated. Results are mean ± standard deviation values.

**Figure 8:**
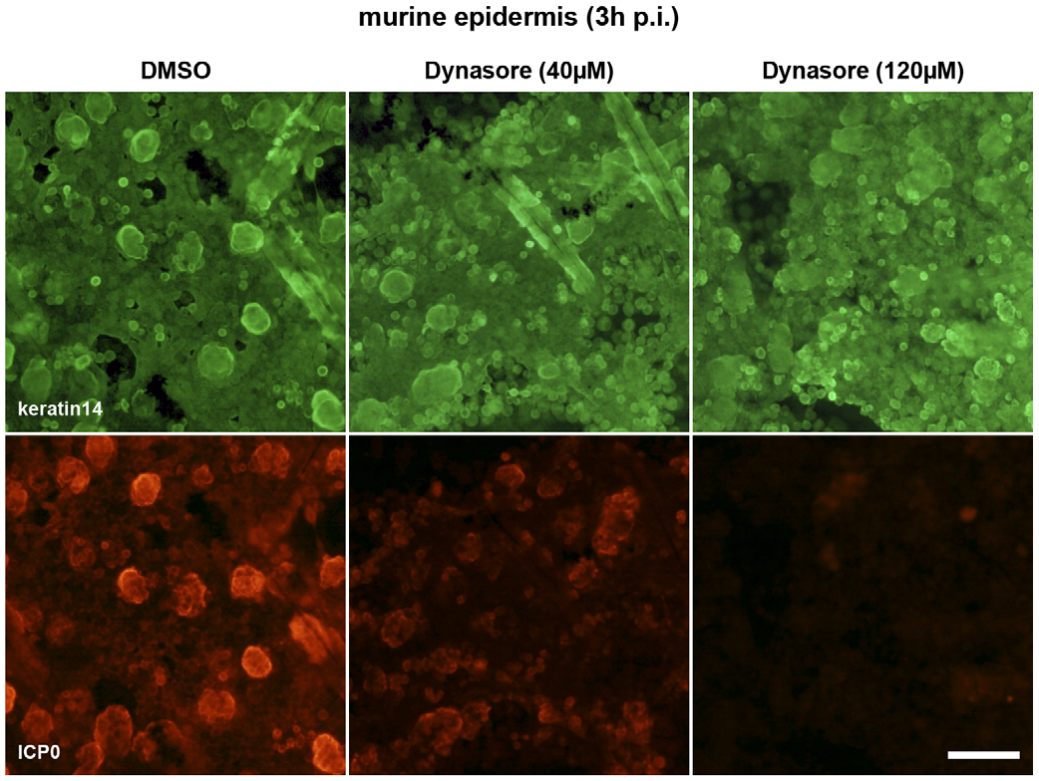
HSV-1 infection of murine epidermis pretreated with dynasore. Epidermal sheets prepared from newborn mouse skin were separated from dermis by dispase II treatment, followed by incubation on 40 or 120 µM dynasore-containing medium or DMSO-containing medium. After 1 h at 37°C HSV-1 was added at 100 PFU/cell. At 3 h p.i. epidermal whole mounts showing the basal keratinocyte layer and developing hair follicles were costained with mouse anti-ICP0 (red) and rabbit anti-keratin 14 (green) visualized with AF555-conjugated anti-mouse (Molecular Probes) and AF488-conjugated anti-rabbit (Molecular Probes), respectively. Single immunofluorescence analyses are shown. Bar, 80 µm.

EM studies were performed to determine how dynasore affected the uptake mechanisms during HSV-1 entry into keratinocytes. As a precondition for the EM studies we tested whether dynasore blocked infection at the high MOIs needed for EM analysis of incoming virus particles. Infection studies with 1500 PFU/cell showed that 120 µM dynasore still blocked infection, while DMSO alone had no effect (Fig. 7B). As expected, the patterns of virus uptake at 10 min p.i. in cells pretreated with DMSO were comparable with those shown for untreated cells with both free capsids and particles in vesicles being observed in the cytoplasm (Fig. 7A a–b, D a–b). In contrast, when primary keratinocytes were pretreated with dynasore we observed virus particles almost exclusively on the outside of the cells at both 10 and 30 min p.i. (Fig. 7A e–h). These particles were predominantly located in invaginations at the plasma membrane, unlike particles that were attached to the cell surface upon incubation for 1 h at 4°C (Fig. 8A c–d). Quantification revealed that there was no increase in the low number of internalized particles in dynasore-treated cells between 10 and 30 min p.i. by which time about 81% of the observed particles had been taken up in control cells (Fig. 7C). Surprisingly, the few internalized particles in dynasore-treated cells included both free capsids underneath the plasma membrane and enveloped particles in vesicles. Dynasore has been reported to stabilize pit formation at the plasma membrane at early and late stages [29]. Therefore, we had assumed that the uptake of particles via fusion of their envelopes with the plasma membrane would not be blocked in dynasore-treated keratinocytes. Since cytoplasmic capsids were more often found in infected HaCaT cells than in primary keratinocytes, we looked for the presence of free capsids in dynasore-treated HaCaT cells at 10 min p.i., by which time approximately 20% of particles in untreated cells would be free capsids in the cytoplasm (Fig. 1C). Surprisingly, as in primary keratinocytes, virus particles were only found on the cell surface in invaginations of the plasma membrane (Fig. 7D, E). Thus, we conclude that dynasore blocks both modes of uptake fusion with the plasma membrane and endocytosis.

Using a recently established protocol for *ex vivo* infection of murine epidermal sheets [16], we investigated whether infection of a target tissue is also dynamin-dependent. Skin from the backs of newborn mice was prepared, and the epidermis was separated from the dermis by dispase treatment. The epidermal sheets were allowed to float on virus-containing medium supplemented with DMSO alone, or with 40 or 120 µM dynasore. At 3 h p.i. costaining of keratin 14 and ICP0 revealed infection throughout the basal layer of keratinocytes in the DMSO-treated epidermis (Fig. 8). In contrast, infection was blocked in a concentration-dependent manner when the epidermal sheets were pretreated with dynasore (Fig. 8). These results support the essential role of dynamin for HSV-1 entry into basal keratinocytes of the epidermis.

In summary, we conclude that dynamin plays an essential role during HSV-1 uptake into keratinocytes.

## Discussion

HSV-1 can use a variety of entry modes all depending on a set of envelope viral glycoproteins [1]. Whether the virus can accomplish more than one entry pathway to infect any particular target cell, is still unclear. In this study, we investigated the entry pathway(s) into human keratinocytes which represent one of the natural target cells for HSV-1. Initial EM studies revealed free capsids underneath the plasma membrane in addition to enveloped virus particles in vesicles suggesting that HSV-1 can enter keratinocytes both by fusion with the plasma membrane and by endocytosis. The challenge is to distinguish whether both or only one of these entry pathways led to productive infection. We initially investigated the impact of endocytic uptake for initiation of infection using a variety of pharmacological inhibitors. In general, the major advantage of pharmacological inhibitors is the short exposure time that delays compensatory responses of the cells. However, the poor specificity of many commonly used drugs can hamper the identification of the precise endocytic pathway involved in virus uptake since they may perturb multiple cellular processes. To minimize this problem we used a range of drugs at concentrations that have been shown to interfere with virus uptake without major side effects. We confirmed that the concentrations of the drugs used in our assays had neither cytotoxic effects nor caused morphological changes in keratinocytes. Our data support endocytic uptake as contributing to HSV-1 entry, although, we are only just beginning to identify the components which characterize the route(s) of uptake.

Our studies in chlorpromazine treated cells showed no effect on the number of infected cells and suggest that clathrin-mediated endocytosis does not play a role in HSV-1 entry into keratinocytes. In contrast, we observed a decreased number of infected cells after treatment with EIPA. EIPA can inhibit the enhanced fluid phase uptake that is associated with particle invagination during macropinocytosis [19]. Macropinocytosis is utilized for entry by a number of pathogens [33], [34], and has been suggested to be involved in Kaposi's sarcoma-associated herpesvirus entry [35]. To further examine the putative role of macropinocytosis in HSV-1 entry we investigated the role of F-actin which is mostly associated with macropinocytic activity using cytochalasin D [33]. Since interference with actin polymerization had only minor effects on infection, our inhibitor studies do not support macropinocytosis as a major uptake mechanism in keratinocytes. This is in line with our previous findings that HSV-1 entry into keratinocytes is independent of Rac1 signaling [16] which participates in the regulation of macropinosome formation [33]. Based on reports showing that EIPA mediates a number of effects on endocytic pathways [20], [36], we conclude that the inhibitory effect of EIPA points in general to the involvement of endocytic uptake but not to macropinocytosis specifically.

A decreased number of infected cells was observed when we analyzed the effects of the microtubule-disrupting agent nocodazole in both HaCaT cells and primary keratinocytes. The importance of the microtubule network for the transport of capsids to the nucleus during HSV-1 entry has been shown previously [37]. Concomitantly, endosomal trafficking also relies on the integrity of the microtubule network [38]. Thus, our studies support a role for microtubules during HSV-1 entry into keratinocytes but do not distinguish between one initiated by fusion with the plasma membrane and releasing capsids into the cytosol or one involving endocytic uptake and vesicle transport.

It has previously been reported that endosomal acidification is required to release HSV-1 after endocytic uptake in keratinocytes [4]. We also observed that lysosomotropic agents such as NH_4_Cl and monensin reduced infection in primary keratinocytes. However, although NH_4_Cl also reduced infectivity in the keratinocyte cell line HaCaT, only minor effects were observed with monensin. These studies suggest that endosomal acidification plays a more prominent role in primary keratinocytes. This is in line with our EM studies which showed that more virus particles were found in vesicles than as free capsids in primary cells as compared to HaCaT cells. Although our results are consistent with the previously described effect of NH_4_Cl, they differ from those described for monensin [4] which may be explained by the different experimental setting.

Taken together our inhibitor studies support pH-dependent endocytic pathway(s) as a route for HSV-1 uptake into keratinocytes leading to productive infection. Interestingly, endocytic uptake seems to be more pronounced in primary keratinocytes than in the keratinocyte cell line highligthing the importance of carrying out studies in primary cells. Although our EM studies suggest that uptake of HSV-1 by fusion with the plasma membrane occurs alongside endocytosis, it remains to be determined whether and to what extent the direct fusion pathway leads to successful infection. The observation that the inhibitors of endocytosis never blocked infection completely but only reduced the number of infected cells or simply delayed infection may be an early indication that fusion at the plasma membrane can also lead to infection.

Our studies revealed a requirement for cholesterol for HSV-1 uptake in keratinocytes. The depletion of cholesterol from the plasma membrane by MβCD resulted in inhibition of infectivity which was slightly stronger in HaCaT cells than in primary keratinocytes. The availability of cholesterol may be different in primary keratinocytes and HaCaT cells. The requirement for cholesterol suggests that lipid rafts may play an essential function in HSV-1 uptake into keratinocytes. Recent studies suggest that lipid rafts act as platforms for HSV-1 entry into Vero cells and mouse melanoma cells expressing either nectin-1 or HVEM involving the interaction of gB with cellular components in the rafts [21]. Interestingly, the HSV-1 receptors nectin-1 and HVEM were not found to be associated with lipid rafts when expressed in mouse melanoma cells. Thus, Bender et al. [21] argued that cholesterol may be required for fusion with the plasma membrane independent of whether the virus receptors are present in lipid rafts or not. Whether nectin-1, a potential HSV-1 receptor in HaCaT cells [39] is localized to lipid rafts in human keratinocytes, is still unknown. Our results suggest that cholesterol is essential during HSV-1 uptake into keratinocytes and we hypothesize that cholesterol supports both fusion with the plasma membrane and endocytic uptake.

In addition to cholesterol our studies demonstrate the essential role of dynamin during HSV-1 entry into keratinocytes. Dynasore, a specific inhibitor of the dynamin GTPase activity [29], [40], blocked HSV-1 infection in HaCaT cells at low concentrations. Interestingly, we observed that infection of primary keratinocytes was less sensitive to dynasore inhibition, requiring levels four times higher to achieve a reduction comparable to that seen in HaCaT cells. This was unexpected since we supposed a more prominent role of endocytic uptake in primary cells, and expected dynamin to be involved in endocytic pathways. The high amount of dynasore was also tested in primary murine neurons where no effect on HSV-1 infection was observed. In principle, dynasore can block endocytic pathways in hippocampal neurons [41]. Thus, dynamin seems to be nonessential for HSV-1 entry into neurons, but plays a major role during the uptake mechanism(s) into keratinocytes. We also examined the requirement for dynamin in murine epidermis using an *ex vivo* infection assay. After treatment of epidermal sheets with dynasore we observed a block of ICP0 expression in the basal keratinocytes suggesting that dynamin also plays a role during entry of the virus into intact tissue. EM studies in primary keratinocytes and HaCaT cells confirmed that neither free capsids nor enveloped particles in vesicles were present inside dynasore-treated cells. The only particles seen were enveloped virions trapped in plasma membrane invaginations at the cell surface. These results suggest that HSV-1 entry is completely dependent on dynamin-mediated pathway(s) which appear to include both early fusion events at the plasma membrane and vesicle scission. HIV, another enveloped virus, has long been assumed to fuse directly at the plasma membrane. Recent findings support HIV entry via endocytosis and suggest a role of dynamin in HIV release from endosomes [42]. The authors argue that the dynamin-dependent fusion with endosomes could rely on the ability of dynamin to regulate actin remodeling and/or associate with membrane-bending proteins which might facilitate endosomal fusion [42]. However, a recent study suggests a role of dynamin in pore expansion following hemifusion [15] which provides a possible reason why HSV-1 fusion at the plasma membrane was blocked by dynasore. Probably, we are only at the beginning of understanding the precise mechanisms underlying dynamin function during viral uptake and that its role is more diverse than presently perceived.

In summary, we suggest that HSV-1 uptake into human keratinocytes involves endocytic pathway(s) and fusion at the plasma membrane, and that both routes are dynamin-mediated and cholesterol-dependent. To understand the underlying mechanisms it will be important to characterize the contribution of nectin-1 and other HSV-1 receptors in human keratinocytes.

## Methods

### Cells, viruses, and plasmids

HaCaT cells [43] were maintained in DMEM (Invitrogen) containing 10% fetal calf serum (FCS) and penicillin (100 IU/ml), streptomycin (100 µg/ml). Primary human foreskin keratinocytes were prepared and cultured on feeder layers as described [44]. In brief, primary human keratinocytes were maintained in keratinocyte culture medium (Ham's F12-DMEM (1∶3); Invitrogen) containing 1.8 mM calcium ions and 10% FCS, penicillin (100 IU/ml), streptomycin (100 µg/ml), adenine (1.8×10^−4^ M), glutamine (2 mM), hydrocortisone (0.5 µg/ml), epidermal growth factor (EGF 10 ng/ml), cholera enterotoxin (10^−5^ M), insulin (5 µg/ml) in the presence of mitomycin C treated 3T3 fibroblasts, strain J2. DMEM/Ham's F-12 (Biochrom) containing 50 µM calcium and 10% calcium-free FCS was used as keratinocyte culture medium to maintain the primary cells under calcium-reduced conditions.

Hippocampal murine neuron cultures were prepared as described [45]. In brief, hippocampi were dissected from embryonic day 9 mice. After treatment with 0.1% trypsin and 150 µg/ml DNAse for 30 min at 37°C, cell suspensions were mechanically dissociated by pipetting and finally centrifuged at 400 g for 5 min. About 20,000 cells were plated on poly-lysine coated coverslips in B27 neurobasal medium supplemented with 1% L-glutamine. Cultures were maintained for about 10 days before infection.

Murine epidermal sheets were taken from back skin of wild-type (C57BL6) newborn mice. At 3 days after birth mice were decapitated and skin pieces of about 15 mm diameter were taken. After incubation for 30 min at 37°C with 5 mg/ml dispase II (Roche) in PBS, the epidermis was washed three times in PBS, gently removed from the underlying dermis as an intact sheet using forceps, and used immediately for infection studies.

Infection studies were performed with purified preparations of HSV-1 wildtype strain 17 as described [46]. In general, virus inoculum was added to the cells at 37°C defining time point 0. In addition, virus was preadsorbed for 1 or 2 h at 4°C as indicated. Virus titers were determined on Vero cells. Pretreatment of virus with 40 µM dynasore or 10 mM MβCD was performed for 30 min at 37°C or room temperature, respectively.

The expression vector encoding eGFP-tagged dynamin1 mutant K44A was obtained from Harvey McMahon (MRC, Cambridge). Plasmid EGFP-C1 (Clontech) was used as control.

### Ethics statement

The preparation of neuronal cells and epidermal sheets from sacrificed animals was carried out in strict accordance with the recommendations of the Guide of Landesamt für Natur, Umwelt and Verbraucherschutz, Nordrhein-Westfalen (Germany). The study was approved by LANUV NRW (Number 8.84-02.05.20.11.058).

### Inhibitor studies

Cytochalasin D and nocodazole (Sigma), and the dynamin inhibitors dynasore (Tocris) and MiTMAB (Calbiochem) were dissolved in dimethyl sulfoxide (DMSO). Methyl-β-cyclodextrin (MβCD) (Sigma) and chlorpromazine (Sigma) were dissolved in water; monensin (Sigma) and 5-(N-Ethyl-N-isopropyl)amiloride (EIPA) (Sigma) were dissolved in ethanol. Cells were treated with the appropriate drugs for 30 min followed by infection at 37°C in the continued presence of the drug. Only MβCD was removed prior to infection by washing the cells with medium three-times. Cholesterol (Sigma) was used to replenish depleted cholesterol in the plasma membrane; cells pretreated with MβCD for 30 min at 37°C were washed three-times, and cholesterol was added for 30 min at 37°C followed by three further washing steps prior to infection. Alexa Fluor 594-conjugated cholera toxin B (Molecular Probes) served as control for the cholesterol-depleting function of MβCD; cells pretreated with MβCD for 30 min at 37°C were washed and incubated for 15 min at 4°C. After addition of AF594-conjugated cholera toxin B, cells were incubated for 10 min at 4°C followed by 10 min at 37°C and fixation. AF488-conjugated transferrin (Molecular Probes) was used as a control for dynasore inhibition. Transferrin was added to dynasore-treated or untreated cells for 15 min at 37°C, and removed from the cell surface prior to fixation by washing with 0.1 M glycine, 150 mM NaCl (pH 2.5) prior to fixation [47].

### Transient expression

For transfection, HaCaT cells were trypsinized, pelleted, washed with PBS and resuspended in Nucleofector solution V (Amaxa). Cells (∼1×10^6^) were transfected with 2 or 4 µg of plasmid in a cuvette, utilizing program U-20 of an Amaxa Nucleofector I. Cells were seeded on coverslips and infected at 22 h posttransfection at 50 PFU/cell for 2 h.

### Immunocytochemistry and antibodies

HaCaT cells and primary keratinocytes grown on coverslips were fixed with 2% formaldehyde in PBS, permeabilized with 0.5% NP-40 and stained. At 2 h p.i. infected cells were visualized by ICP0 staining with mouse anti-ICP0 (monoclonal antibody 11060) [48], diluted 1∶60. Capsid protein VP5 was visualized with monoclonal antibody DM165 [49], diluted 1∶200. For immunostaining of epidermal wholemounts, murine epidermis was fixed with 3.4% formaldehyde for 2 h, washed two times with PBS, blocked with 0.5% milk powder, 0.25% gelatin from cold water fish skin, 0.5% Triton X-100 in 0.2% PBS-Tween 20 for 1 h, and then incubated overnight with mouse anti-ICP0 (monoclonal antibody 11060) [48] diluted 1∶60 and rabbit polyclonal anti-mouse keratin 14 (AF64, Covance) diluted 1∶100,000 followed by overnight incubation with secondary antibodies at room temperature. Staining of F-actin was performed with tetramethylrhodamine isothiocyanate (TRITC)-conjugated phalloidin (Sigma). Microscopy was performed using a Zeiss Axiophot, a Zeiss LSM 510 and a Leica DM IRB/E microscope linked to a Leica TCS-SP/5 confocal unit. Images were aquired using Adobe Photoshop, version CS2.

The effects of dynamin1 mutant K44A were quantified by counting about 300 transfected cells visualized by GFP fluorescence in three independent experiments and calculating the number of infected cells visualized by ICP0 staining.

### Electron microscopy

Infected cells were prepared for electron microscopy as described [50]. Thin sections were cut, stained with uranyl acetate and lead citrate, and analyzed in a JEOL 1200 EX II transmission electron microscope. For quantification we examined sections of 0.1 µm. In each section we analyzed 80–100 cells with every third cell showing at least one virus particle. For each time point and experiment sections of about 30 cells with 60–90 virus particles in total were evaluated.
